# Laparoscopic treatment of biliary peritonitis following nonoperative management of blunt liver trauma

**DOI:** 10.1186/1749-7922-5-26

**Published:** 2010-09-15

**Authors:** Ettore Marzano, Edoardo Rosso, Elie Oussoultzoglou, Olivier Collange, Philippe Bachellier, Patrick Pessaux

**Affiliations:** 1 Pôle des Pathologies Digestives, Hépatiques et de la Transplantation. Hôpital de Hautepierre Universitaires de Strasbourg, Avenue Molière, 67098 Strasbourg Cedex, France; 2 Department of Anesthesiology. Hôpital de Hautepierre Universitaires de Strasbourg, Avenue Molière, 67098 Strasbourg Cedex, France

## Abstract

**Introduction:**

Nonoperative management (NOM) of hemodynamically stable patients with blunt hepatic injuries is considered the current standard of care. However, it is associated with several in-hospital complications. In selected cases laparoscopy could be proposed as diagnostic and therapeutic means.

**Case report:**

A 28 years-old male was admitted in the Emergency Unit following a motor vehicle crash. CT-scan showed an isolated stade II hepatic injury at the level of the segment IV. Firstly a NOM was decided. Laparoscopic exploration was then performed at day 4 due to a biliary peritonitis. Intraoperative trans-cystic duct cholangiography showed a biliary leaks of left hepatic biliary tract, involving sectioral pedicle to segment III. Cholecystectomy, trans-cystic biliary drainage, application of surgical tissue sealing patch and abdominal drainage were performed. Postoperative outcome was uneventful, with fast patient recovery.

**Conclusion:**

Laparoscopy has gained a role as diagnostic and therapeutic means in treatment of complications following NOM of blunt liver trauma. This approach seems feasible and safety, with satisfactory postoperative outcome.

## Background

Nowadays nonoperative management of blunt hepatic injuries is considered the treatment of choice in about 70% of cases. This attitude lead to appearance of otherwise unknown complications including bleeding, biliary, infectious and abdominal compartement syndrome. In selected cases, laparoscopy could be considered a valid option to treat these complications.

### Introduction

Nonoperative management (NOM) of hemodynamically stable patients with blunt hepatic injuries is considered as the current standard of care [1,2]. Recent series reported that approximately 70% of patients with blunt liver injuries can be treated nonoperatively, with no hepatic-related mortality [3]. However, nonoperative treatment has been associated with several in-hospital complications, including bleeding, biliary, infectious and abdominal compartement syndrome. In this scenario, laparoscopy as gained a role as diagnostic and therapeutic means with favourable results [4,5]. Nevertheless, its application still remain under-proposed.

### Case report

**Figure 1:**
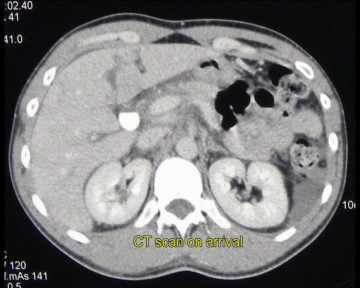
**CT-scan at arrival**.

**Figure 2:**
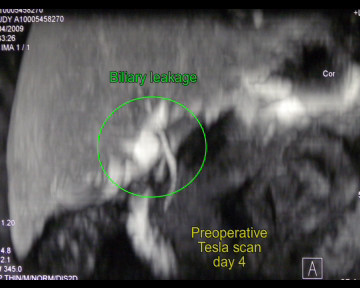
**Preoperative Teslascan**.

**Figure 3:**
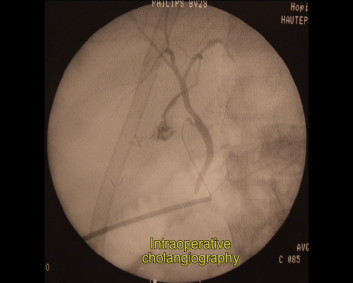
**Intraoperative cholangiography**.

A 28 years-old male was admitted in the Emergency Unit following a motor vehicle crash. The patient was hemodynamically stable (blood pressure = 110/70 mmHg; cardiac frequency = 95/min) and conscious (Glasgow coma score = 15). The clinical examination showed an abdominal distension and diffuse pain. FAST echography revealed a moderate peritoneal effusion. Total-body CT scan was performed, which showed an isolated stade II [6] hepatic injury at the level of the segment IV (fig 1). Haemoglobin at admission was 12.3 g/dl (normal range 13-18 g/dl) and remained stable at 11.7 g/dl 6 hours after. NOM was decided. Four days after the admission, due to the appearance of an inflammatory response on blood test - CRP 101 mg/dl (normal <4 mg/dl) white cells 15.6 10*9/L (normal range 4.10-10.50 10*9/L) - and the persistence of abdominal pain, an hepatic MR with TESLASCAN (fig 2) was performed which showed a biliary leaks originating from left liver. Laparoscopic exploration revealed an intense biliary peritonitis. Liquid sample was performed. Hepatic exploration confirmed the presence of a liver fracture of segment IV without signs of active bleeding. Cholecystectomy followed by a trans-cystic cholangiography (fig 3) showed a biliary leaks of left hepatic biliary tract, involving sectioral pedicle to segment III. Hemostatic and tissue sealing (Nycomed TachoSil^®^) surgical patch was applied on liver injury, in order to minimized biliary spillage. Two intra-abdominal and a trans-cystic biliary drains were inserted in view to drain abdominal cavity and biliary tree, respectively (Additional file 1). Postoperative outcome was uneventful and patient was discharged at postoperative day 18^th^.

## Conclusions

Liver related morbidity after NOM of blunt liver injury is reported within 12% rate in most series [2,5,7]. Hepatic related complications usually consisted in: bleeding, biliary, hepatic abscess or necrosis, and development of abdominal compartment syndrome.

Concerning biliary complications, bile duct injury, development of bilioma and biliary peritonitis were mostly described [7,8]. Multimodality management consisting of, radiological drainage, endoscopic stenting and surgery is frequently performed. Surgical treatment is reserved to patients who developed generalised biliary peritonitis or in case of failure of less invasive procedures. In this scenario laparoscopic surgery has become a valid option as diagnostic and therapeutic means. In some referral centres delayed laparoscopy is even routinely proposed [8]. Thus laparoscopy should not be considered as a failure of NOM but as a part of this therapeutic strategy. In our experience laparoscopy was performed because of appearance of an inflammatory response on blood test and diffused peritonitis at clinical examination.

Finally, utilisation of hemostatic and tissue sealing agent (Nycomed TachoSil^®^) seams to give an effective control of biliary fistula. In our case the biliary leakage was successfully treated by application of the surgical patch on the liver fracture after scrupulous lavage of the hepatic surface. Utilisation of such a device in elective liver surgery is well known and its hemostatic properties are already reported [9]. Afterwards, tissue sealing characteristics were observed in repairing air leakage following pulmonary resection [10]. Moreover, bile leaks reduction after application of Tachosil surgical patch, was observed in a retrospective series about adult split liver transplantation [11] and resective hepatic surgery [12]. Probably, a real tissue repairing and reinforcing properties with construction of a neo hepatic glissonien capsule could be supposed. In our experience the patient did not develop any biliary fistula documented by drainage output and any endoscopic complementary procedure was necessary to treat the biliary injury.

In conclusion laparoscopy and application of Tachosil surgical patch was an efficient and definitive treatment of a biliary complication following NOM of blunt liver injury.

## Consent

Written informed consent was obtained from the patient for publication of this case report and accompanying images. A copy of the written consent is available for review by the Editor-in-Chief of this journal

## Competing interests

The authors declare that they have no competing interests.

## Authors' contributions

Conception and design: ER, PP.

Collection and assembly of data: EM, EO, OC.

Data analysis and interpretation: EM, EO, OC.

Manuscript writing: EM, ER.

All authors read and approved the final manuscript.

## Supplementary Material

Additional file 1 **Video of surgical procedure** Biliary peritonitis following blunt liver trauma. Click here for file
